# SEOM guidelines for cervical cancer

**DOI:** 10.1007/s12094-015-1452-2

**Published:** 2015-12-09

**Authors:** A. Oaknin, M. J. Rubio, A. Redondo, A. De Juan, J. F. Cueva Bañuelos, M. Gil-Martin, E. Ortega, A. Garcia-Arias, A. Gonzalez-Martin, I. Bover

**Affiliations:** Medical Oncology Department, Vall d’Hebron University Hospital, Vall d’Hebron Institute of Oncology (VHIO), Ps Vall d’Hebron, 119-129, 08035 Barcelona, Spain; Hospital Universitario Reina Sofía de Córdoba, Córdoba, Spain; Hospital Universitario la Paz, Madrid, Spain; Hospital Universitario Marqués de Valdecilla, Santander, Spain; Complejo Hospitalario Universitario de Santiago, Santiago de Compostela, Spain; Hospital Durán i Reynals (ICO), Barcelona, Spain; Hospital Universitari Arnau de Villanova de Lleida, Lleida, Spain; Institut Català d’Oncologia, Hospital de Sant Joan Despí-Moisès Broggi, Barcelona, Spain; MD Anderson Cancer Center, Madrid, Spain; Hospital Son Llatzer, Palma de Mallorca, Spain

**Keywords:** Cervical cancer, Human papilloma virus, Clinical stage

## Abstract

Cervical cancer (CC) is the second most common cancer worldwide, strongly linked to high-risk human papilloma virus infection. Although screening programs have led to a relevant reduction in the incidence and mortality due to CC in developed countries, it is still an important cause of mortality in undeveloped countries. Clinical stage is still the most relevant prognostic factor. In early stages, the primary treatment is surgery or radiotherapy, whereas concomitant chemo-radiotherapy is the conventional approach in locally advanced stages. In the setting of recurrent or metastatic CC, for the first time ever, the combination of chemotherapy plus bevacizumab prolongs the overall survival beyond 12 months. Therefore, this regimen is considered by most of the oncologist a new standard of care for metastatic/recurrent CC.

## Introduction

Cervical cancer represents a unique disease in the field of oncology due to the presence of well-established risk factors, a long pre-invasive period which allows the use of screening tests, a very well-established etiologic agent, namely HPV infection and the availability of effective preventive vaccination against this infection.

Every year 500,000 new cases and 250,000 deaths occur worldwide being the majority of them reported in developing countries because of the lack of access to screening programs based on Pap smear. By contrast, in the industrialized countries due to the implementation of the population screening campaigns, the incidence of cervical cancer has dramatically decreased [1].

The majority of cervical cancer cases are comprised by squamous cell carcinoma, although to date, adenocarcinoma can reach the 25 % of new cases. Human papilloma virus (HPV) is a sexually transmitted DNA virus responsible for almost all cervical cancers regardless of histology. More than 200 subtypes of HPV have been identified, there being only 18 oncogenic subtypes. Among these oncogenic subtypes, the serotypes 16 and 18 are responsible for almost 70–90 % of the cases. Although the majority of women will be able to clear the HPV infection during the 2 years after its onset, some of them will develop a persistence of the virus in the cervical epithelium, leading to the development of a pre-malignant lesion and eventually an invasive cervical cancer 15–20 years after the first infection. Vaccines against HPV 16 and 18 have demonstrated a clear reduction in the development of pre-invasive lesions. However, the rate of implementation is variable worldwide and even within the same country. Therefore, screening with HPV detection with or without Pap smear is still needed following international consensus guidelines. Unfortunately, even with well-established screening programs, women will develop cervical cancer. Treatment approaches for women affected by invasive cervical cancer are presented in these guidelines.

### Guidelines methods

Under the auspices of the Spanish Society of Medical Oncology (SEOM) and with the cooperation of Grupo Español de Investigación en Cáncer de Ovario (GEICO), a number of experts in the field together with two coordinators were designed to develop this clinical practice and evidence-based guideline. Different levels of recommendation and evidence were associated with each conclusion of the guideline according to the US Agency for Health Research and Quality scoring.

## Diagnosis and staging in cervical cancer

Invasive cervical cancer usually presents as irregular or post-coital bleeding and/or foul-smelling discharge; nevertheless, most of the early-stage cervical cancer patients have no symptoms at diagnosis.

Cervical cancer diagnosis is performed through cervical cytology, colposcopically guided biopsy or gross palpable lesion biopsy. The aforementioned procedures allow for accurate diagnosis.

The recommended staging system is based on the Federation International Gynaecology and Obstetrics (FIGO) classification (Table 1). The FIGO staging is a clinical classification based on tumor size, vaginal and/or parametrial involvement and bladder/rectum tumor extension determined by pelvic examination. Interestingly, only chest radiography, intravenous pyelography, cystoscopy and proctoscopy (if apparent bladder or rectal involvement) are permitted to better define the FIGO stage. Since most of cervical cancer cases occur in developing countries with poor resources, the FIGO Committee has decided not to modify the clinical staging by the incorporation of a work-up with pelvic MRI and PET/CT [2]. Nevertheless, in most of the developed countries, both imaging tests comprise the initial diagnosis procedures. Pelvic MRI is the preference for treatment planning, especially for fertility-sparing surgery. PET/CT scan is widely used to determine the lymph nodes (pelvic and para-aortic) status, namely pathologic or not. However, in some training sites, surgical staging, as extraperitoneal laparoscopic para-aortic lymph node dissection, is the preferred approach to determine the lymph nodes status, since the pathological analysis is the most reliable assay. The lymph node evaluation allows us to tailor the radiotherapy treatment (category 2B of recommendation). Currently, an ongoing phase III clinical study will determine the best staging procedure [3–5]. Sentinel lymph node mapping is another surgical approach to improve early cervical cancer staging with promising results that requires further evaluation (Level of evidence IIB) [6]. A laboratory test including renal function evaluation must also be performed.

**Table 1:** 2009 FIGO classification

I: The carcinoma is strictly confined to the cervix (extension to the uterine corpus should be disregarded)
IA: Invasive cancer identified only microscopically. Invasion is limited to measured stromal invasion with a maximum depth of 5mm^a^ and no wider than 7 mm
IA1: Measured invasion of stroma ≤3 mm in depth and ≤7 mm width
IA2: Measured invasion of stroma >3 mm and <5 mm in depth and ≤7 mm width
IB: Clinical lesions confined to the cervix, or preclinical lesions greater than stage IA
IB1: Clinical lesions no greater than 4 cm in size
IB2: Clinical lesions >4 cm in size
II: The carcinoma extends beyond the uterus, but has not extended onto the pelvic wall or to the lower third of vagina
IIA: Involvement of up to the upper two-third of the vagina. No obvious parametrial involvement
IIA1: Clinically visible lesion ≤4 cm
IIA2: Clinically visible lesion >4 cm
IIB: Obvious parametrial involvement but not onto the pelvic sidewall
III: The carcinoma has extended onto the pelvic sidewall. On rectal examination, there is no cancer-free space between the tumor and pelvic sidewall. The tumor involves the lower third of the vagina. All cases of hydronephrosis or non-functioning kidney should be included unless they are known to be due to other causes
IIIA: Involvement of the lower vagina but no extension onto pelvic sidewall
IIIB: Extension onto the pelvic sidewall, or hydronephrosis/non-functioning kidney
IV: The carcinoma has extended beyond the true pelvis or has clinically involved the mucosa of the bladder and/or rectum
IVA: Spread to adjacent pelvic organs
IVB: Spread to distant organs

^a^The depth of invasion should not be more than 5 mm taken from the base of the epithelium, either surface of glandular, from which it originates. Vascular space invasion should not alter the staging

FIGO staging guidelines were most recently updated in 2009 (Table 1). The most relevant changes were the following ones: stage 0 was deleted because it is a pre-invasive lesion and stage IIA was split based on tumor size (less or greater than 4 cm as maximum diameter). It is remarkable that neither lymphovascular space invasion (LVSI) nor lymph node metastases are included in the FIGO classification.

## Treatment

### Early stages

After careful clinical evaluation and staging, the primary treatment of early-stage cervical cancer is either surgery or radiotherapy (RT). The treatment approach is determined by the FIGO stage (Table 2).

**Table 2:** CC treatment algorithm (early stages)

IA1	If patient desires fertility: conization If patient does not: simple hysterectomy
IA2	Hysterectomy ± pelvic lymphadenectomy ± para-aortic lymphadenectomy radiotherapy If patient desires fertility: trachelectomy + pelvic lymphadenectomy ± para-aortic lymphadenectomy
IB1	Radical hysterectomy with pelvic ± para-aortic lymphadenectomy radiotherapy
IB2	Cisplatin-based chemotherapy concurrent with external beam radiotherapy + vaginal brachytherapy
IIA1	Radical hysterectomy with pelvic ± para-aortic lymphadenectomy radiotherapy
IIA2	Cisplatin-based chemotherapy concurrent with external beam radiotherapy + vaginal brachytherapy

#### Stage IA1

Treatment options for stage IA1 without LVSI may include simple hysterectomy. Cone biopsy could be considered for patients who desire to preserve fertility. Lymphadenectomy is not necessary because the risk of node metastasis is less than 1 %. If positive margins following conization or LVSI are present, modified radical hysterectomy with pelvic lymph node dissection is recommended (Level of evidence IIb; Grade of recommendation B) [7]; however, if preserve fertility is desired, radical trachelectomy and pelvic lymphadenectomy could be performed in very well-selected patients.

#### Stage IA2

Stage IA2 tumors can be treated with radical hysterectomy or radical trachelectomy (for patients who wish to preserve their fertility) and pelvic lymph node dissection with (or without) para-aortic lymph node sampling (Level of evidence IIB; Grade of recommendation B). Para-aortic node dissection is indicated for patients with known or suspected pelvic nodal disease (Level of evidence IIB; Grade of recommendation B). For patients who are not suitable for surgery or who refuse the procedure, pelvic radiation with brachytherapy is an option.

#### Stage IB1/IIA1

For patients with stage IB1 or IIA1 disease, the surgical approach is the election, including radical hysterectomy and bilateral pelvic lymph node dissection with or without para-aortic lymph node sampling. Para-aortic node dissection is indicated for patients with large tumors and suspected or known pelvic nodal disease. Recently, a prospective randomized study showed that there are no significant differences in terms of both recurrence rate and overall survival among patients with stage IB1–IIA1 cervical cancer undergoing simple extrafascial hysterectomy or radical hysterectomy, and morbidity is proportional to the extent of radicality [8]. For selected patients, namely those with stage IB1 and tumor size less or equal to 2 cm, who desire fertility preservation, radical trachelectomy and pelvic lymph node dissection with (or without) para-aortic lymph node sampling can be considered [9]. In cases of inoperable patients, combining pelvic RT and brachytherapy is an alternative therapy [10]. The role of concurrent cisplatin-containing chemotherapy in early stages is still underevaluation; therefore, this option has to be taken with caution.

It is worth mentioning that the pathologic results of a surgical procedure provide reliable information about risk factors in order to consider adjuvant treatment. A prospective study in patients with node-negative stage IB identified intermediate-risk factors for recurrence such as tumor diameter >4 cm, deep cervical stromal invasion and positive LVSI (Sedlis criteria). In the presence of two of them, adjuvant treatment with radiotherapy should be recommended as it has shown a statistical benefit in terms of progression-free survival [11] (Level of evidence IIa; Grade of recommendation B). In addition, The Intergroup Trial 0107 showed that patients with early-stage disease who underwent surgery and had high-risk factors in the surgical specimen such as positive lymph nodes, positive margins and/or microscopic parametrial involvement obtained an statistically significant benefit in overall survival (OS) from concurrent pelvic radiation plus chemotherapy (cisplatin/5-FU) as a complementary treatment compared to observation only (Level of evidence Ib; Category of recommendation A) [12].

### Locally advanced stages (LACC)

This category has traditionally included patients with stage IIB–IVA disease. However, currently, patients with IB2 and IIA2 stages are also included in this category.

The National Cancer Institute announced in 1999 to consider concurrent chemo-radiation as the new standard of care for patients diagnosed with LACC. This announcement was made based on the results of five randomized clinical trials showing a statically significant benefit in overall survival for the patients receiving concomitant treatment. These positive results were later confirmed by a meta-analysis based on 18 trials [12]. These results demonstrated a 6 % and 8 % improvement in absolute 5-year survival and disease-free survival with chemo-radiotherapy, respectively. In addition, a decreasing relative effect of chemo-radiotherapy on survival with increasing tumor stage was observed, with estimated absolute survival benefits of 10 % (stage Ia to IIA), 7 % (stage IIB) and 3 % (stage III to IVA) at 5 years. The most common regimen used in concurrent treatment is cisplatin monotherapy at a dose of 40 mg/m^2^ (maximum 70 mg as total dose) on a weekly schedule. The optimal doses of radiation therapy are 80–90 Gy to the target volume, delivered by both external beam radiotherapy (EBRT) and brachytherapy (BT). Concurrent chemo-radiotherapy should not exceed 8 weeks as a longer period of time results in worse tumor control and survival. A critical issue is the volume of RT, which is usually guided by pelvic and para-aortic nodes involvement. Therefore, imaging studies (including PET/CT) and/or surgical staging is strongly recommended for stages ≥IB2. Primary cisplatin-based chemo-radiotherapy is the treatment of choice in locally advanced cervical cancer (Level of evidence Ia; Recommendation level A).

In the aforementioned meta-analysis [12], patients who received adjuvant chemotherapy after chemo-radiation presented a significant risk reduction in death, with an absolute benefit of 19 % at 5 years. Nevertheless, these results are based only on two clinical trials, so they must be taken with caution. In addition, Dueñas et al. [13] also showed a significant improvement in PFS and OS among women who received two cycles of cisplatin plus gemcitabine following concurrent chemo-radiation. However, in this treatment arm, women also received gemcitabine added to weekly cisplatin during radiation. So, we do not know whether the benefit is the result of the adjuvant treatment, the concurrent treatment or both. The role of adjuvant chemotherapy merits further investigation. This issue is being addressed in an international randomized study OUTBACK trial, sponsored by the Gynecologic Cancer Intergroup (clinicalTrials.gov identifier: NCT01414608).

The role of neoadjuvant chemotherapy in LACC remains controversial since we do not have yet data from a randomized clinical trial that compares this approach followed by surgery to concurrent chemo-radiation [14]. Currently, we are waiting for the EORTC 55994 clinical trial results that studies neoadjuvant chemotherapy in FIGO stages IB2, IIA and IIB. In addition, the phase III INTERLACE trial (clinicalTrials.gov identifier: NCT01566240) is analyzing the role of neoadjuvant chemotherapy before concurrent chemo-radiation compared with concurrent chemo-radiation alone in stages IB2–IVA. To date, neoadjuvant chemotherapy before either standard concurrent chemo-radiation or radical surgery is not a standard approach (Level of evidence IIa; Category of recommendation C).

## Locoregional or metastatic recurrent disease

### Local/regional therapy

Recurrent or persistent disease after initial treatment should be confirmed by a biopsy before starting a new treatment, mainly in case of isolated and/or small lesions. Local/regional treatment options for recurrence depend on the initial treatment modality.

- Patients who have not undergone previous radiotherapy (RT) (or with recurrences outside of the RT field): The preferred therapy includes external RT and platinum-based chemotherapy, with or without brachytherapy [15] (Level of evidence IIa; Grade of recommendation B).
- Patients with central pelvic recurrence after RT: They should be evaluated for pelvic exenteration (Level of evidence IIb; Grade of recommendation B). This procedure usually comprises besides the hysterectomy, a cystectomy as well as resection of rectum and vagina. In very well-selected patients, exenteration is associated with a survival rate of approximately 50 % at 5 years [16]. In order to achieve a curative intent, clear margins are mandatory. Unfortunately, this objective is only achieved in half of the procedures (Level of evidence IIb; Grade of recommendation B). Although MRI and PET/CT are widely used to diagnose cervical cancer recurrences, neither techniques identify the precise extent of the disease, which ultimately leads to cancellation of the surgery in the operating room. Age should not be an issue to establish exenteration indication since retrospective studies have not shown more morbidity/mortality in older patients.
  Although exenteration is the common surgical approach in patients with previous RT, in selected patients with isolated and small central lesions, hysterectomy or brachytherapy may be an option (Level of evidence IV; Grade of recommendation C). The role of intraoperative radiotherapy (IORT) has been evaluated in several retrospective studies, but with confusing results. Thus, there is no evidence to recommend this treatment routinely [17] (Level of evidence III; Grade of recommendation C).
- Patients with non-central recurrence after RT: There are different options depending on the location of the lesions and the performance status of the patient: resection (with or without IORT), chemotherapy or best supportive care (Level of evidence IV; Grade of recommendation C).

### Metastatic disease

The implementation of concurrent chemo-radiation as standard of care in LACC has dramatically decreased the risk of recurrence; nevertheless, we are still witnessing recurrent disease. The risk of recurrence ranges from 16 to 30 % in early stages up to 70 % in LACC. Most relapses occur within the first 2 years after diagnosis, and 50–60 % of patients will have disease outside the pelvis; thus, palliative systemic chemotherapy is an important therapeutic option for these patients.

Cisplatin had been for a long period of time the most active cytotoxic in the treatment of cervical cancer, with median overall survival (OS) less than 7 months [18]. Because of these poor results, different ways were sought to improve them. The first maneuver was to increase the dose of cisplatin. The results from a GOG (Gynecologic Oncology Group) randomized phase III study established the dose of cisplatin at 50 mg/m^2^ every 3 weeks as a standard treatment in recurrent or metastatic cancer of cervix since higher doses were associated with a greater toxicity without improvement in OS (Level of evidence Ib; Grade of recommendation A) [2].

A remarkable change in the metastatic/recurrent cervical cancer treatment occurred with the publication of results from two important randomized clinical trials, the GOG#169 and GOG#179 [19, 20]. Both studies compared cisplatin, single agent, to cisplatin in combination with either paclitaxel or topotecan, respectively. The combination with paclitaxel was superior in terms of response rate (RR) and progression-free survival (PFS) but not in OS (8.8 vs 9.7 months) (Evidence level 1B). However, the combination of cisplatin with topotecan showed statically significant improvement in all their end points: RR, PFS and OS. In December 2006, FDA approved the combination of cisplatin/topotecan in the treatment of advanced cervical cancer (Level of evidence Ib; Grade of recommendation B).

Later on, to clarify which was the most effective cisplatin doublet, the phase III trial, GOG#204, was developed. Three chemotherapies regimens based on cisplatin were compared to the GOG standard of care, cisplatin/paclitaxel (CP) [21]. Even though no statically significant differences were reached, the combination of CP shows a positive tendency in terms of PFS and OS, which reinforced its role as standard of care. Unfortunately, the median OS was still around 12 months. Therefore, it was unmet need in the treatment of this disease. In this scenario, the anti-angiogenic therapy in cervical cancer started to be studied due to the association between angiogenesis and invasive cervical cancer phenotype. The GOG #227 evaluated the anti-VEGF agent, bevacizumab among 46 women with persistent or recurrent squamous cell cervical carcinoma. Exciting results were reached with a RR of 11 %, PFS at 6 months of 24 % and OS of 7.29 months [22]. These results led to GOG240, a phase III, randomized clinical trial of 452 women with metastatic or recurrent cervical cancer. Patients were randomized to chemotherapy (cisplatin 50 mg/m^2^ plus paclitaxel 135 or 175 mg/m^2^ or topotecan 0.75 mg/m^2^ d1-3 plus paclitaxel 175 mg/m^2^ d1) with or without bevacizumab 15 mg/kg [23]. As compared with cisplatin and paclitaxel, the topotecan–paclitaxel backbone did not significantly impact OS (HR 1.2, 98 % CI 0.82–1.76). We can infer that the non-cisplatin doublet is neither superior to cisplatin regimen nor inferior as well. However, the incorporation of bevacizumab significantly improved OS compared to chemotherapy alone (17 vs. 13.3 months, HR 0.71, 97 % CI 0.54–0.94) and PFS (8.2 vs. 5.9 months; HR 0.67, 95 % CI 0.54–0.82). The response rate (RR) was significantly higher in patients treated with bevacizumab (48 vs. 36 %; *p* = 0.00807).

The results achieved with the combination of bevacizumab plus chemotherapy are clinically meaningful. It is the first clinical trial in metastatic cervical cancer that shows OS greater than 12 months, actually 17 months.

These results led to expedite approval of bevacizumab in the treatment of recurrent/metastatic cervical cancer by the FDA in August 2014 and later in March 2015 by the EMA. To date, this regimen should be considered the new standard of care in recurrent and/or metastatic cervical cancer (Level of evidence Ib; Grade of recommendation A).

### Surveillance

No definitive agreement exists on the best post-treatment surveillance. The recommended surveillance is based on the patient’s risk of recurrence [23].

It is recommended having a clinical visit including a pelvic examination with cervical/vaginal cytology every 3–6 months for the first 2 years, every 6 months for the next 3 years and then annually. Patients with high-risk disease can be assessed more frequently (every 3 months for the first 2 years) than patients with low-risk disease (every 6 months) (Level of evidence III; Grade of recommendation B).

The probability of diagnosing a cervical cancer recurrence based on only cervical cytology is quite low, therefore a proper clinical evaluation together with a high index of suspicion are required.

Imaging tests are not routinely recommended for surveillance. A CT or a PET/CT scan should be performed as clinically indicated in patients with symptoms or findings that are suspicious for recurrence. In patients at high risk of local–regional (central or para-aortic) failure, a PET/CT scan may be useful for detecting asymptomatic disease that is potentially curable. There is no consensus about how often a PET/CT should be done in this high-risk group at least once a year would be recommended [24] (Level of evidence 2; Grade of recommendation B).

There are no blood tumor markers recommended for the surveillance in these patients.
